# Chemical biology probes of mammalian GLUT structure and function

**DOI:** 10.1042/BCJ20170677

**Published:** 2018-11-20

**Authors:** Geoffrey D. Holman

**Affiliations:** Department of Biology and Biochemistry, University of Bath, Bath BA2 7AY, U.K.

**Keywords:** GLUT proteins, GLUT4 translocation, insulin action

## Abstract

The structure and function of glucose transporters of the mammalian GLUT family of proteins has been studied over many decades, and the proteins have fascinated numerous research groups over this time. This interest is related to the importance of the GLUTs as archetypical membrane transport facilitators, as key limiters of the supply of glucose to cell metabolism, as targets of cell insulin and exercise signalling and of regulated membrane traffic, and as potential drug targets to combat cancer and metabolic diseases such as type 2 diabetes and obesity. This review focusses on the use of chemical biology approaches and sugar analogue probes to study these important proteins.

## Introduction

This review is based on the 2017 Randle Lecture delivered at Bath University at a Biochemical Society meeting on ‘Insulin and exercise signalling for glucose homeostasis and metabolic health’. First, I would like to pay tribute to Sir Philip Randle. Sir Philip's contributions to the field of metabolic control in heath, obesity and type 2 diabetes have been immense. His ideas on the cross-talk between fat and glucose metabolism are still influential today. This review focusses on the first step in glucose metabolism, that is glucose transport, without which cells would not be able to supply metabolic processes with carbohydrate substrates. Sir Philip described the glucose transport step as a site of feedback control of carbohydrate metabolism under conditions in which fat was available as a substrate [1]. This fundamental process has been studied over the many decades using a huge range of techniques and approaches and in this review, I focus on just five of these decades. My career of obsession with, and addiction to, the subject began in 1970 during my PhD work at Southampton University where we worked on sugar analogues that interact with glucose transporters.

The single technique that characterises most of our studies is that of chemical biology. We did not describe our analogous studies in the 1970s by this name. This name came into common use much later [2,3]. According to Wikipedia, ‘Chemical biology is a scientific discipline spanning the fields of chemistry, biology, and physics. It involves the application of chemical techniques, tools, and analyses, and often compounds produced through synthetic chemistry, to the study and manipulation of biological systems’. As chemical biology underpinned many of my early studies, and later (from 1976) those of my group at Bath University, I focus this review on these approaches realising, of course, that many researchers in the glucose transport research field have used a wide range of approaches ranging from cell and signalling biology to genetic manipulation and pharmacological interventions.

So, what are the glucose transporters that have so fascinated researchers over many decades and what are the key questions concerning their structure and function and regulation? I focus on the mammalian glucose transporters (GLUTs) that catalyse (facilitate) passive movement of glucose down concentration gradients [4]. These gradients are usually from the blood system to the cell interior, but in the liver these gradients can be from the cell to the blood stream. The GLUT family of transport proteins thereby cooperatively function to supply glucose in the direction needed for cell metabolic processes while maintaining a remarkably constant blood glucose level (5 mM after fasting).

The GLUT1 transporter is present in high amounts in human erythrocytes and, because of the relative ease of working with these cells, it has been most studied from the structure related to function perspective [5]. It has a *K*_m_ value for glucose influx (∼2 mM) which is significantly lower than blood glucose levels [6–8]. The large amounts of the protein (∼5% of the membrane protein) were an important factor in its purification. A peptide sequence was obtained and used to identify a cDNA clone and ultimately the DNA sequence [5]. GLUT1 is still the only endogenous GLUT that has been purified to homogeneity and which can be identified as a Coomassie-staining protein on an SDS–PAGE gel. GLUT1 is present in most human cells and is abundant at the blood–brain barrier [9].

GLUT2 is present in the liver and pancreas and presumably other cells of the endoderm lineage [10]. GLUT3 is mainly present in the brain [11]. A GLUT3 variant (GLUT14) has also been found in the genome as a duplicon of GLUT3 [12], but is essentially uncharacterised and of unknown function and tissue distribution, although there is some disease association [13]. GLUT2 and GLUT3 have *K*_m_ values for glucose transport that are higher and lower, respectively, than fasting blood glucose reflecting the functions of these proteins in supplying glucose (GLUT2) and rapidly and avidly removing glucose (GLUT3) from the circulation. GLUT4 is present mainly in the insulin-sensitive tissues of adipose, heart and skeletal muscle [14,15]. Its *K*_m_ for glucose substrate is close to the fasting blood glucose level and this is unchanged by insulin action [16], which instead leads to an increase in glucose transport by increasing GLUT4 translocation to the cell surface of target cells [17].

Genome sequencing has identified 14 mammalian glucose transporter-like proteins which have been divided into three phylogenetically distinct groups [18]. GLUT1–4 and GLUT14 constitute Class 1. The main substrate is glucose with much lower affinity for fructose [19]. GLUTs 5, 7, 9 and 11 are Class 2 transporters. GLUT5 is a fructose transporter with higher affinity for fructose than glucose [20] and is abundant in small intestine, kidney and sperm with lower levels in fat and skeletal muscle [21]. GLUT 7 and 11 transport glucose and fructose with approximately equal affinity [22,23]. GLUT9 has affinity for fructose in the physiological range but also transports urate, and this is probably the physiological substrate [22,24]. The Class 3 transporters include GLUTs 6, 8, 10, 12 and 13, and their tissue distributions, functions and specificities have not been extensively characterised. The preferred substrates for this group may not be glucose, and this is clearly the case for GLUT13, which is a myo-inositol transporter [18,19].

## The structure and catalytic function of the GLUT proteins

### GLUT hydrogen bonding to substrates and spatial requirements of interacting substrate analogues

**Figure 1.**
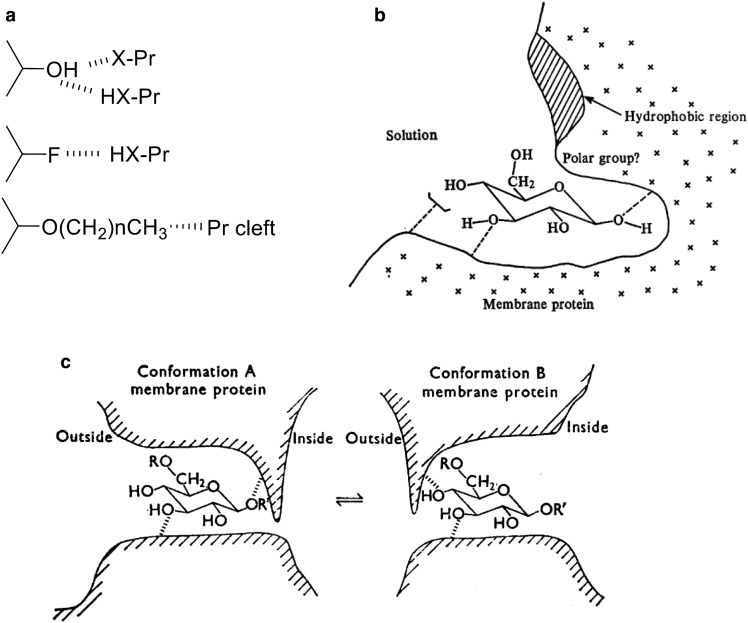
Use of glucose analogues to explore the GLUT-binding site structure. (**a**) Hydroxyls may H-bond to GLUT side chains through electronegative membrane groups to the H of the glucose OH, or from a protonated membrane group to the O of the glucose OH. A fluorine group can only H-bond with a protonated membrane group. Alky substitutions (for example, methyl and propyl) can probe the spatial limitations around the binding site cleft. (**b**) Hydrogen bonds occur to C1β-O, C3-O, C4-OH as indicated. H-bonding to C6-O occurs with an additional hydrophobic interaction with a membrane group which was suggested to be a tyrosine or tryptophan. Figure from Figure 4 of ref. [25]. (**c**) If R=R′=H, both conformations of the membrane are possible, and transport occurs. If R=propyl and R′=H, binding can occur only with conformation A. If R=H and R′=propyl, binding can occur only with conformation B. The approach to the binding site alternates between outward-facing and inward-facing clefts which polarise the sugar with C1-OH leading and C4/C6-OH trailing. Figure from Figure 1 of ref. [26].

During my PhD work with John Barnett at Southampton University, we examined the hydrogen bonding and spatial requirements for erythrocyte GLUT1 with a range of sugar analogues. This was, of course, before the identification of the protein that catalysed this process. The fluorine substitution to replace the glucose hydroxyls (–OH) gives information on the direction of the H-bonds (Figure 1a). If the bond is from the protein side-chain to the electronegative O of the –OH, then the electronegative F will substitute. If the H-bond involves this H of the –OH, then it will not. We found H-bonding to the C1-β-O of glucose and to the C3-O, C4-OH, C6-O positions (Figure 1b). 2-Deoxy-glucose bound as well as glucose and the C2-OH was considered to be unimportant in glucose-binding and the transport catalysis process [25].

Alkyl substitutions into the glucose hydroxyls were also informative. An O-alkyl-substituted glucose analogue can act as an inhibitor of the transport of substrate if there is sufficient space around the binding site in this position of the substitution. A C1 β-*O*-methyl glucose analogue is not an inhibitor of substrate transport when added to the outside of the erythrocyte. This suggests that the relatively small methyl group is not tolerated by GLUT1 and that there is a close approach between the C1-OH position of glucose and the protein cleft on the external surface (Figure 1b,c). However, we were able to show that there is good tolerance of bulky substitutions around C4 and C6 at the external site of GLUT1, for example 4-*O*-propyl-d-glucose, 6-*O*-propyl-d-galactose, 4,6-*O*-ethylidene-d-glucose and maltose bound well from outside the cell. These analogues bind well but are not transported, suggesting that bulk around the C4–C6 prevents a conformational change associated with transport [25]. We also found that alkyl substitutions at C6 even led to increased affinity. Since H-bonding and a hydrophobic interaction occur, we suggested [25] that ‘either a tryptophan or a tyrosine residue suitably placed with respect to C6 of the sugar could fulfil both these functions’.

We also investigated the binding to the inside site using alkyl substitutions into C1 β-OH. C1-*O*-alkyl glucose compounds do not appear to enter the cell through the GLUT. However, when we allowed a long chain glucoside (propyl-β-d-glucoside) long enough to enter through the membrane lipid, then it was a good inhibitor from the inside site of the transporter. We found this for erythrocyte GLUT1 during my PhD studies [26,27] and much later after I moved to Bath, when Bill Rees looked at the same side-specific inhibitors for adipocyte GLUT4 [28,29]. These studies led us to propose an alternating binding-cleft model (Figure 1c) for the transport process in which the sugar bound to the transporter in a polarised manner, with C1-OH orientated inwards towards the cell interior and C4/C6-OH trailing. This model was published in our 1973 and 1975 papers for the *Biochemical Journal* and has since been supported by a range of studies (reviewed in ref. [4]) and most recently in the crystal structural studies on the GLUTs (described below).

### Photoaffinity labelling of GLUTs and evidence for large structural changes in transport catalysis

**Figure 2.**
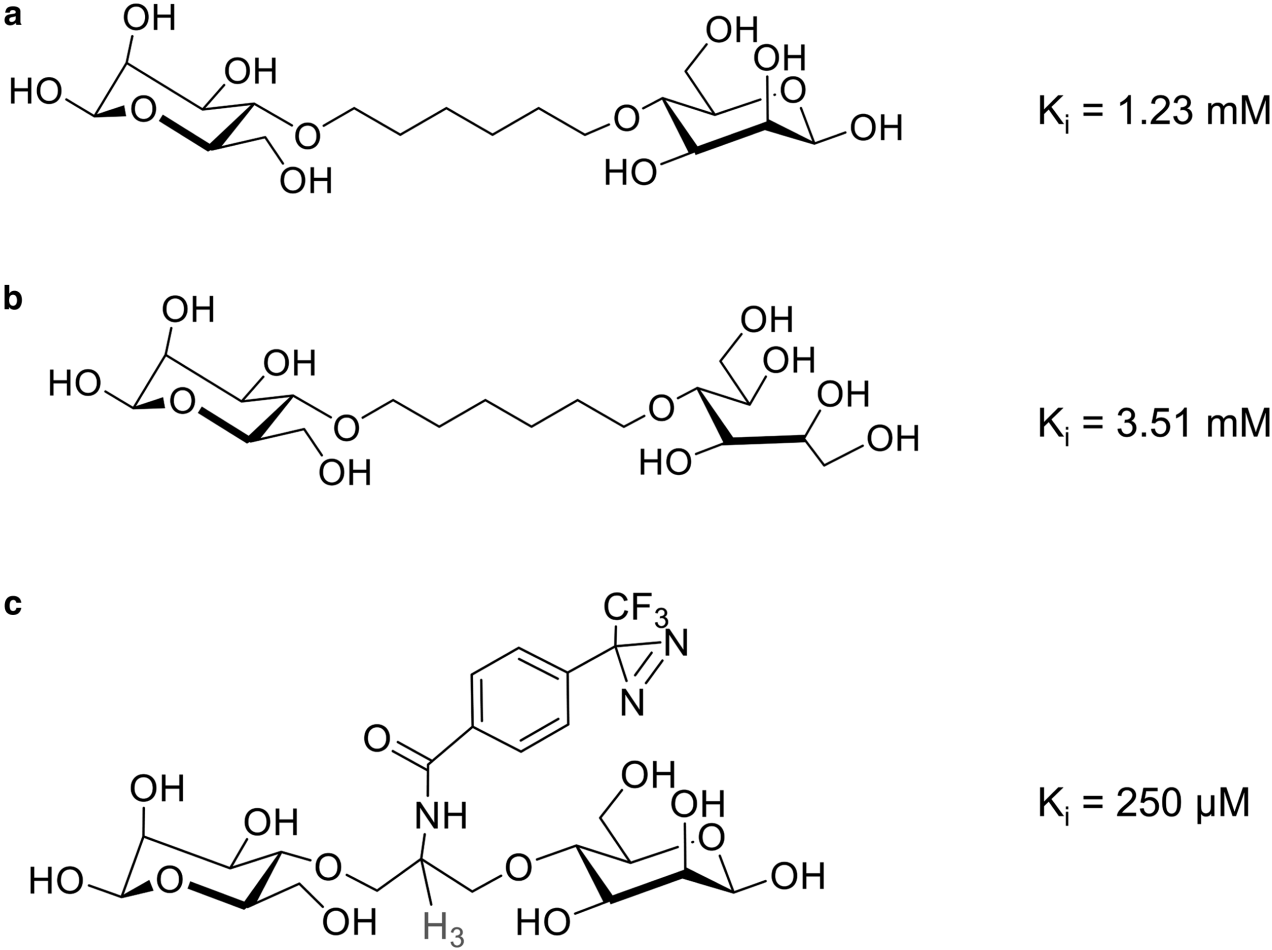
Bis-mannose analogues. Bis-mannose compounds were produced by linking two d-mannose moieties through the C4-OH position, as analogue studies had revealed space around this region of the binding cleft in GLUT1 and GLUT4 and an enhancement in affinity from hydrophobic substitutions around C4/C6. (**a**) A hexane linker has been used; the *K*_i_ for interaction GLUT4 was 1.23 mM. (**b**) The hexane-linked d-mannose has been half reduced which opens the ring; the affinity for interaction with GLUT4 is reduced by 2-fold. In (**c**), a propylamine-linked bis-mannose has been substituted with a photoreactive group to produce ATB-BMPA. The associated increase in hydrophobicity leads to increased affinity; *K*_i_ for interaction with GLUT4 was 250 µM. Tritium was introduced into the linking propylamine.

The information on the shape of the binding cleft at the exofacial surface of the GLUT1 and GLUT4 transporters gained from analogue studies was useful in the design and development of photoaffinity labels. These were developed to specifically interact at the exofacial site of these transporters. A range of bis-sugars with the link between the two hexose moieties through C4-OH (Figure 2) was developed [30–32]. The compounds are very hydrophilic because of the bis-hexose hydroxyls and sufficiently bulky to block any conformational change that occurs after binding. These factors restrict the binding to the external site of the transporter. These molecules have a double chance of binding at the external cleft through the important C1-OH. We found that if we half reduced this molecule, so that one of the C1-OHs is converted to CH_2_OH, then the affinity dropped by one half [33] (Figure 2a,b). This is clearly attributable to just halving the glucose moieties in a single molecule. At one stage, we wondered whether the transporter might allow multiple occupancy. However, if this occurred, we anticipated that the bis-sugar structure would result in an affinity enhancement greater than the 2-fold effect observed. We obtained high affinity analogues by varying the substituent in the middle linker between the C4-OH positions [33]. We think that these affinity-enhancing substitutions interact with the hydrophobic surface around C4/C6 described above (Figure 1b). The linker bridge also allowed us to introduce tritium as indicated (Figure 2c). We started work on the bis-mannose compound as mannose (the C2-OH glucose epimer) has only slightly less affinity than glucose, as there is no H-bonding to C2-OH [25,28]. The affinity of the GLUT1 and GLUT4 transporters for the bis-mannose compounds is quite good compared with the parent sugar (over a 10-fold enhancement in affinity compared with the parent compound mannose). Several types of photolabelling substituents were added to the bridge, including azides, benzophenone and diazirine groups, and the diazirine type of substitution was found to be most effective. The diazirine photolabelling moiety was used to synthesise ATB-BMPA (Figure 2c).

We have used bis-hexose photolabels to narrow down the labelling site in GLUT1 to a region in TM8/9 [34,35]. This was worked out from a series of chemical and enzymatic digests of the transporter into smaller fragments. This exofacial binding site was distinguishable from a site labelled by UV cross-linking with cytochalasin B, a side-specific competitive inhibitor of the endofacial site [7]. Chemical and enzymatic cleavage after labelling revealed a different series of fragments arising from cytochalasin B-labelled transporter (TM10/11) to those parts of the protein labelled by the exofacial-specific photolabels. These fragmentation data suggested that the C-terminal half of the protein contained a large portion of the glucose binding sections and that the exofacial and endofacial ligand binding sites were distinct [35].

The interaction with substrates and photolabels is altered by mutations in several regions but most notably by mutations in TM7 and TM10/11. We carried out some GLUT1 mutation studies with the Masato Kasuga's group [36–38]. We identified that mutation of tyrosine 293 locked the transporter into the outward-facing conformation. The mutated protein interacted with the bis-mannose photolabels but not with cytochalasin B. We proposed that this tyrosine was critical for closing the binding site around the trailing end of the glucose molecule as it enters the outside binding cleft [36] as postulated in the earlier hexose analogue studies (Figure 1b). Tyrosine 292 and 293 are both important in GLUT1-mediated glucose transport as revealed by other mutagenesis studies, including cysteine scanning mutagenesis [39–41]. We worked with Annetta Schurmann and Hans Joost on the adjacent amino acids, serine 294 and threonine 295 (GLUT1 numbering) of GLUT4 [42]. The serine 294 and threonine 295 mutations also locked of the transporter into a stable outward-facing conformation with good exofacial ligand binding but no cytochalasin B binding and markedly reduced glucose transport. A threonine 295 mutation to methionine has been found to be a human mutation occurring in a form of epilepsy [43].Therefore, it appears that the entire sequence of residues from Tyr292 to Thr295 (GLUT1 numbering) may have a role in opening and closing access at the exofacial side of the GLUT transporters.

We found that a substitution of a proline 385 with isoleucine (but not glycine) in TM10 of GLUT1 [44] reduced the exofacial ligand binding, but retained the cytochalasin B binding. In this paper, we suggested that TM10 was a flexible region that allowed TM7, 8 to move against TM10,11 [44]. In addition, it has been shown that mutation of GLUT1 tryptophan 388 is important for transport and the binding of the inside-site-specific ligand cytochalasin B [45]. Thus, mutations in the top half of TM7 or the bottom half of TM10 have opposite effects, either locking the transporter into an outward-facing conformation or an inward-facing conformation, respectively. These predictions are markedly consistent with the now known crystal structure of the GLUT transporters (below).

Over more recent years, many studies on mutations into GLUT1 (including large cysteine and alanine scanning mutagenesis studies) have identified residues within both the N- and C-terminal halves of the protein, and in internal helical loops of GLUT1, that have been shown to alter function [19,46–50]. Our studies were directed towards amino acids in the TM7–TM11 regions as this is where we had mapped the binding sites that bound photolabels.

Changes in exposure of transporter proteins to protease digestion have been very informative in studies on membrane proteins. We found that this approach is particularly insightful in studies on GLUT1. When the exofacial photolabel is bound outside, there is a large reduction in trypsin digestion [32]. Conversely, cytochalasin B binding increases the exposure to proteases, including trypsin and thermolysin [51,52]. These data suggest that a major conformational change has occurred when ligands bind either outside or inside. This change in conformation is large enough to alter access to proteases which cleave at sites located at the internal solution face of GLUT1. These protein regions may be lifted upward and away from the protease when the external ligand binds. This could be consistent with a component of an ‘elevator’ type of mechanism [53] in the conformational change between outward- and inward-facing sites.

Evidence for stable substructures that are associated with the binding sites of the protein has been obtained from studies on GLUT1 constructs expressed in the baculovirus/Sf9 system. The full-length transporter is labelled well in this system, both by bis-mannose photolabels and by UV cross-linking of cytochalasin B [54]. When either the C-terminal domain or the N-terminal domain is expressed separately in this system, there is no labelling. However, if cells are doubly transfected with virus coding for these two separate N- and C-domain constructs, then the expressed protein domains find each other in the membrane, form a stable aggregated structure which is then functional and binds the labelling compounds. The identified labelling site is still in the C-terminal domain, but the N-terminal domain supports its labelling.

### GLUT alternating conformations revealed by crystallography

As described above, a combination of analogue studies, labelling studies, mutagenesis and sub-domain constructs of GLUT1 led to the following proposals:

- There is polarisation of the sugar in the binding site with C1-OH facing inside and C4–C6 trailing.
- Mapping studies using photoaffinity labelling techniques have led to a focus on helices 7–11 exofacial substrate-binding region, with the exofacial labelled site mapping to helices 7/9 and the endofacial site mapping to helices 10–11.
- We proposed, mainly based on mutagenesis and some modelling studies, that movement of the top of TM7-8 relative to the bottom of TM10-11 facilitates alternate opening and closing of exofacial and endofacial sites.
- Photolabelling and mutagenesis studies suggested that binding sites could be locked into either an outward-facing (bis-mannose binding) or an inward-facing site (cytochalasin B binding) but not both simultaneously.
- There are stable substructures in the protein that move relative to each other to facilitate transport.

Gwyn Gould and I summarised much of these data and hypotheses in our review in the *Biochemical Journal* in 1993 [4]. So how does this collection of data, arising from what could be described as chemical biology techniques, compare with the now known crystal structures of the GLUT proteins?

**Figure 3.**
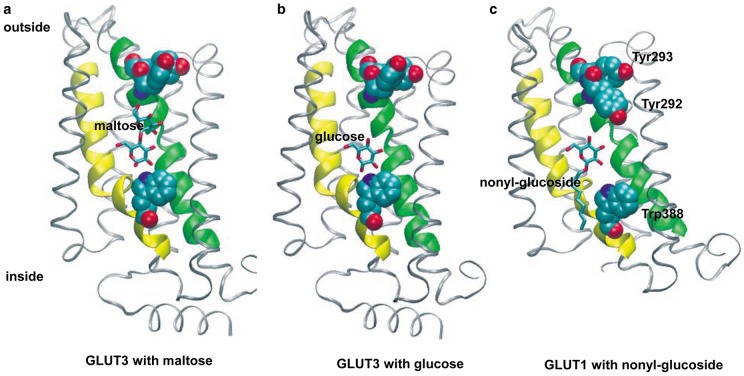
Role of TM7B and TM10A residues of GLUTs in alternating binding site occlusion. Only the C-terminal sections are shown as viewed from the centres of the proteins. TM7 is highlighted in green, TM10 in yellow. (**a**) GLUT3 is in a partially outside-open state with maltose (from PDB: 4ZWB). (**b**) GLUT3 is in a partially outside-open state with glucose (from PDB: 4ZW9). In (**c**), GLUT1 is in an inside-open state with nonyl-β-d-glucoside (from PDB: 4PYB). These structures are described in detail in ref. [56]. In all three cases, the central glucose moiety is in the same position with C1-OH leading inward and C4/C6-OH trailing. In (**a**), the trailing glucose moiety of maltose is above the central glucose. In (**c**), the alkyl chain extends below the central glucose and trails out of the inside-open binding site. The sugar polarity is therefore preserved in the outside-open and the inside-open conformations and is consistent with the predictions based on glucose analogue studies (Figure 1c). Both TM7 and 10 have a linked break in their helix. This allows regions TM7B and TM10A to simultaneously rotate to switch between the outside-open (**b**) and inside-open (**c**) conformations. Tyrosine 292, tyrosine 293 and tryptophan 388 (GLUT1 numbering; GLUT3 numbering is −2 at these positions) are highlighted as space-filling structures to illustrate their movement between the two conformations and their role in site occlusion.

In 2014, Nieng Yan and colleagues described the crystal structure of GLUT1 bound to nonyl-β-d-glucoside [55]. This structure was locked in an inward-facing conformation. This was followed in 2015 by a crystal structure of GLUT3 from the Yan group [56]. Obtaining the GLUT3 structures was a particularly impressive achievement. Structures with GLUT3 bound to glucose or to the disaccharide maltose were obtained. Two different maltose-bound GLUT3 structures were obtained, a partially open structure (as in Figure 3a) and a more fully open structure. In addition, crystal structures of GLUT1 with cytochalasin B bound to an inward-facing conformation have been obtained by Kapoor et al. [57].

The structure of GLUT1 with nonyl-β-d-glucoside bound to the central substrate site revealed a cleft which is open to the inside solution of the cell, with the cleft to the outside clearly closed down and inaccessible (Figure 3c). In contrast, the structure of GLUT3 with exofacially bound maltose (both the open occluded and open structures) had the opposite arrangement of the structural domains around the leading glucose in the substrate site, with a cleft only open to the outside solution and accommodating the trailing glucose moiety in the disaccharide (Figure 3a). The leading glucose moiety occupied the deep centre of the structure. In a separate crystal structure with bound glucose, the glucose (Figure 3b) occupied the same central location as the leading glucose moiety in the maltose structure and in the bound nonyl-β-d-glucoside structure (Figure 3c) indicating localised protein side-chain movement around the centrally located glucose-binding site.

The central position of the bound glucose moiety is essentially the same in all three structures (maltose bound, glucose bound and *n*-nonyl-β-d-glucoside bound in Figure 3a–c, respectively), and it is the protein that is changing shape around the bound ligand (comparing Figure 3a,b with Figure 3c). These crystallographic data are remarkably consistent with the alternating sugar-binding-cleft hypothesis for the glucose transport mechanism that was revealed by analogue studies some 40 years earlier [26,27]. The mechanism of alternate binding-cleft opening has an element of rigid-body rotation of the flexible C-terminal half relative to relatively immobile N-terminal half of the protein. The N-terminal half acts as a platform on which the conformational changes in the C-terminal half occur [56]. In addition, changes in the internal helical linker regions occur that may limit the extent of the conformational changes [56]. In addition, Deng et al. [56] now emphasise the importance in the mechanism of transport catalysis of localised movement of domains and interacting side chains in the C-terminal half of the protein, and particularly with the movement of TM7 and TM8 relative to TM10 and TM11. There are clear changes in the position of helices that must feature in the transition between outward-open and inward-open sites. TM7 and TM10 both have centrally kinked-shapes so that when they move the substrate-binding cavity opens to the outside or the inside. To close the outside site and open the inside site, the top of helix 7 (TM7B) and the bottom of helix 10 (TM10A) twist downward relative to the membrane axis (Figure 3). As described by Deng et al. [56], the segments TM7B and TM10A are structurally related as they occur in equivalent positions in inverted repeats of the helix-bundles TM7-9 and TM10-12. Such inverted repeats arise from gene duplication and fusion and are thought to be favourable for reducing the free-energy required for the conformational change associated with transport, such that little more energy input is required for transport than that associated with substrate binding and release [58,59].

The new crystallographic data are highly supportive of a mechanism for transport in which the catalytic site has alternate access to either the outside or the inside of the cell, but not to both of these simultaneously [60]. However, the complete catalytic cycle for the alternating access model requires that changes (which are similar to those which occur with substrate bound) occur in the absence of substrate. So far, these have not been obtained for the class 1 GLUTs. However, there is evidence for the unliganded forms in outward- and inward-facing forms from other crystal structures including GLUT5 and bacterial GLUT-like transporters [61–63].

Deng et al. [56] suggest that the region to which the sugar binds in the C-terminal half is quite hydrophobic in comparison with the N-terminal half and that the sugar-binding site. Comparisons of crystal structures from Nieng Yan's group [56], and illustrated in Figure 3, reveal that movements of hydrophobic groups such as tyrosine and tryptophan residues are involved in shutting off the glucose cleft either from the external solution (Tyr 292 and 293, GLUT1 numbering) or from the internal solution (Trp 388, GLUT1 numbering) as predicted from the mutagenesis studies described above.

The availability of crystal structures for the GLUTs is a tremendous advance and will pave the way for more studies that reveal the dynamics of the transport catalysis process and provide information that will be useful in the design of GLUT-specific inhibitors. However, it is historically interesting to look back on the early chemical biology experiments as they came close to providing quite accurate insights into the catalytic mechanism for glucose transporters.

## Use of chemical biology tools in studying GLUT translocation to the cell surface of adipose cells

**Figure 4.**
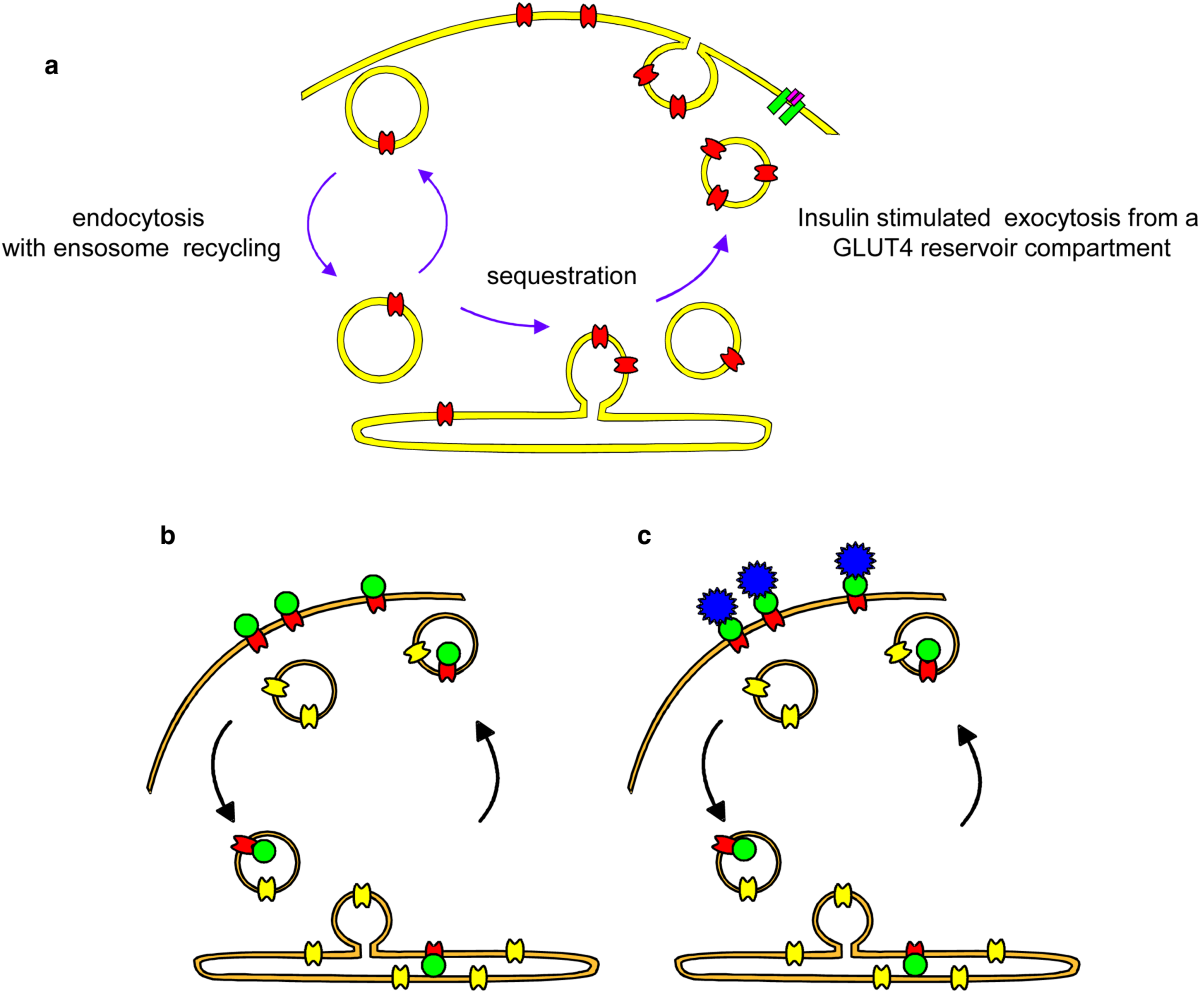
Translocation of GLUT in response to insulin action. The bis-hexose photolabels have been used to investigate the movement of GLUT4 from the cell surface to intracellular compartments and back again. (**a**) The figure is based on the translocation hypothesis of refs [17,64]. The tritiated version of the bis-mannose photolabel, ATB-BMPA, was used to show that GLUT4 (red) continually recycled even in the presence of insulin and that insulin increased the rate of exocytosis from its sequestered reservoir compartment. Modelling suggested that endosome recycling occurred but was not altered by insulin action. GLUT4 can tag biotinylated photolabel (green in **b**) and rates of trafficking can be determined by quenching the surface signal with extracellular avidin addition (blue in **c**) and then detecting the remaining internal GLUT4. This avoids use of subcellular fractionation to separate the plasma membrane from intracellular membranes.

One of the ideas behind the development of the impermeant GLUT photolabel was to tag only the GLUT4 transporter residing at the plasma membrane of the cell and not that residing on any internal membrane vesicles. We planned to use the photolabel to test the GLUT4 translocation hypothesis described independently by the groups of Sam Cushman [17] and Tetsuru Kono [64] in 1980 (Figure 4a). After this model was proposed, there were several features of this model that needed to be tested. Initially, we used a bis-mannose benzophenone compound to show that transporter that was tagged at the cell surface could move into the low density microsome fraction of the cell [31]. We then started a collaboration with Sam Cushman's group to use the photolabelling approach to more quantitatively examine aspects of translocation [65]. With some improvements in the label that involved the introduction of the diazirine photoreactive compound ATB-BMPA (Figure 2c), and with the development of GLUT1- and GLUT4-specific antibodies, we were able to specifically isolate each of these transporters and show that GLUT4 was 10 times more abundant than GLUT1 in rat adipocytes [65]. Shinobu Satoh in Sam Cushman's group was able to follow time courses for GLUT4 movement by using the difficult technique of subcellular fractionation to separate the plasma membrane from intracellular membranes [66]. This fractionation was done on multiple fractions arising from different time points in the kinetic assays. The movement of the GLUT4 out of the plasma membrane and into the low density microsome fraction of membranes (and back again) was followed.

With the photolabelling approach, we were able to show that the GLUT4 recycled even in the continuous presence of insulin. This continuous recycling was a bit of a surprise as it was initially considered that GLUT4 stayed in the surface membrane until insulin was removed from its receptor. Instead, we found that GLUT4 recycled between the plasma membrane and its intracellular reservoir compartment both in the basal and in the insulin-stimulated states and that the exocytosis rate was increased 10-fold by insulin action [66]. This 10-fold activation of exocytosis was sufficient to account for most of the insulin stimulation of GLUT4 net translocation to the surface and with the associated increase in glucose transport activity that occurred after an insulin stimulation of the adipose cells.

The kinetic analysis of translocation revealed that the movement of GLUT4 was somewhat complex and multiple compartments and steps were involved. Simple equations based on just two compartments (the surface and a single internal membrane compartment) did not really fit all the combined data well. The equations are fine for analytical purposes. However, when the data from separate measurements (for example, movement out and movement in) are put together, some inconsistencies emerge. We found that the initial exocytosis of GLUT4 during insulin stimulation from the basal state to the stimulated state was more rapid than the exocytosis we could measure when the system was at steady state and GLUT4 was continuously recycling. We therefore suggested that at least two internal membrane compartments are involved and that insulin-stimulated exit from a specialised reservoir compartment was faster than return to the cell surface by recycling from endosomes (Figure 4a) [67]. Subsequent work by many laboratories resulted in the consideration of several variations in the multiple-compartment GLUT4 recycling ideas. The compartments through which GLUT4 transits included the trans-Golgi, the ER, the retromer system and the ESCRT pre-lysosomal compartment [68–73]. The sorting of GLUT4 between these compartments suggests that GLUT4 is somewhat unique and may traverse many compartments. However, at steady state, a large proportion to the total internal GLUT4 resides in specialised vesicles (the GLUT4-sequestered reservoir compartment, Figure 4a) that contain only IRAP and VAMP2 [68,72,74], and it is these vesicles that are rapidly recruited to and fuse with the plasma membrane in response to insulin signalling.

## Use of biotinylated GLUT photolabels

**Figure 5.**
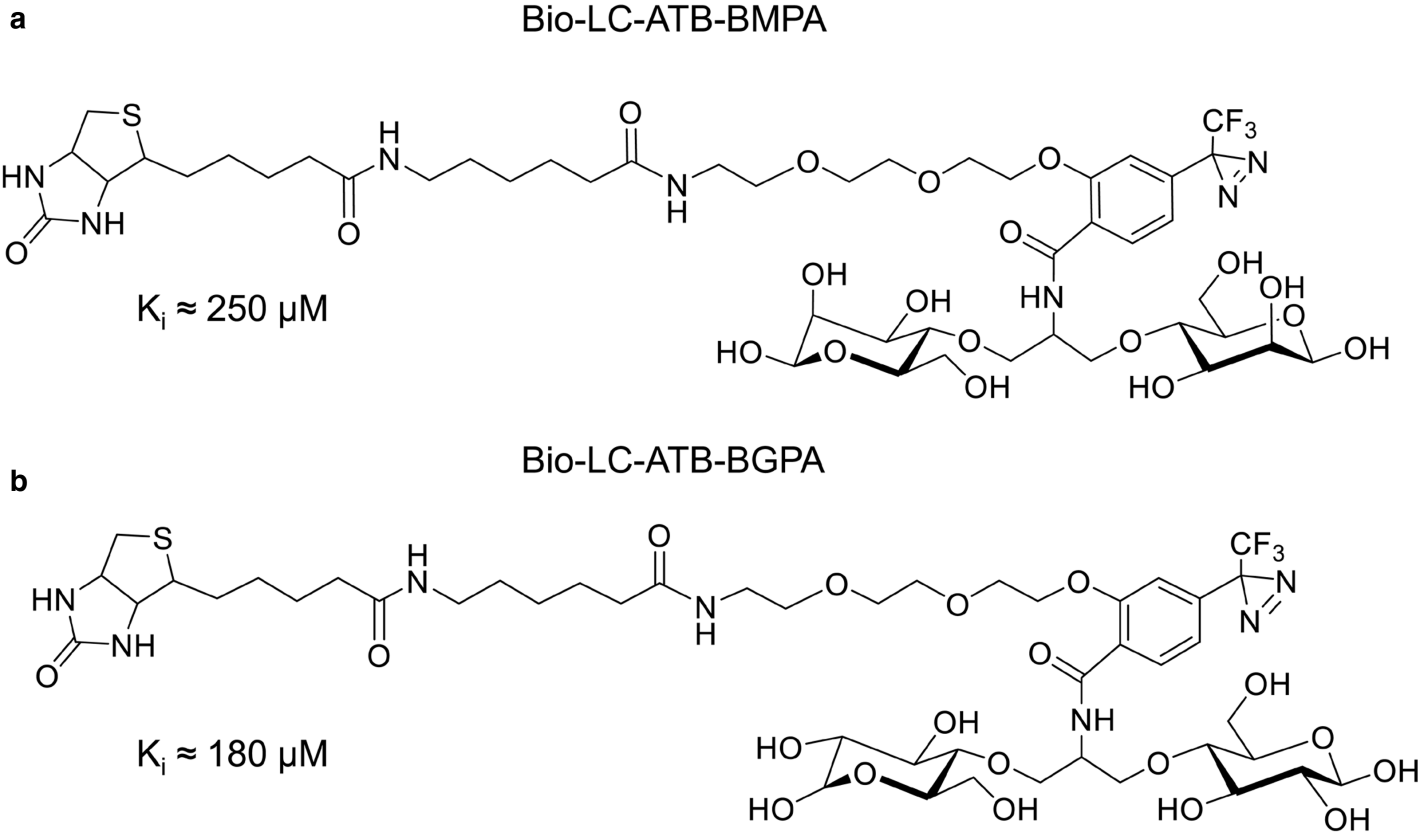
Biotinylated bis-hexoses. A biotin substitution onto the bridge between two hexoses can be introduced without any loss of affinity compared with the non-biotinylated ATB-BMPA. This is consistent with the exofacial binding cleft being deep and open enough to accommodate the large substituent. The bis-glucose (**b**) compound has only slightly higher affinity than the bis-mannose compound (**a**).

Because of the difficulties associated with synthesising the tritiated bis-mannose compound using several mCi of tritiated compound under semi-dark conditions, we eventually decided to synthesise biotinylated versions of the bis-hexose photolabels. Introducing the spacer chain and the bulky group into a biotinylated version of the photolabel (Figure 5a) does not reduce affinity compared with the tritiated compound. This is consistent with the sugar-binding site fitting around the front leading end of the hexopyranose ring and recognising mainly the hydroxyl at C1 and C3. We initially synthesised the biotinylated bis-mannose compound and, several years later, the biotinylated bis-glucose compound (Figure 5b). As expected, the increase in affinity with bis-glucose instead of bis-mannose was only slight (from 220 to 180 µM) [75], but any improvement in affinity is useful as it reduces the amount of reagent needed to achieve a strong signal on blots using the chemiluminescent streptavidin-peroxidase system.

**Figure 6.**
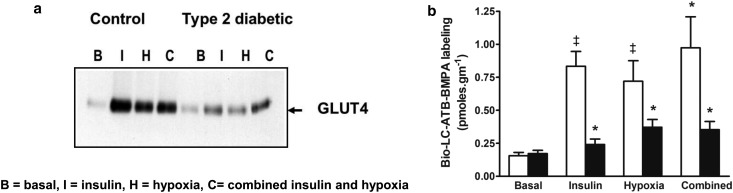
Cell-surface photolabelling of human muscle. (**a**) Type 2 diabetic subjects have consistently lower cell-surface GLUT4 as detected by photoaffinity labelling with Bio-LC-ATB-BMPA. The GLUT4 is tagged with biotin, separated by SDS–PAGE, and blotted with GLUT4 antibody and the signal is quantified (**b**). Insulin, hypoxia and a combined treatment all increase cell-surface GLUT4 above basal levels. These stimulations are reduced in type 2 diabetes (black bars in **b**), but hypoxia and a combined treatment give partial rescue of the impaired insulin response. Data from ref. [76].

In collaboration with Juleen Zierath's group at the Karolinska Institute in Stockholm, we used the biotinylated bis-mannose photolabel to tag the GLUT4 that is exposed at the surface of skeletal muscle from type 2 diabetic and control subjects (Figure 6). In type 2 diabetic subjects, there is a large reduction in the insulin stimulation of cell-surface GLUT4. In the diabetic subjects, GLUT4 labelling is increased by hypoxia treatment and the combined treatment with insulin and hypoxia (Figure 6a,b) [76] or by the combination of insulin and the AMPK activator AICAR [77]. The AMPK signalling system is part of a key pathway that responds to hypoxia, AICAR, metformin, exercise and muscle contraction. It still seems rather unclear whether this ‘exercise’ pathway can fully compensate for the defective insulin stimulation that occurs in type 2 diabetic subjects, but pharmaceutical companies are investigating stimulators of this pathway as a possible means of therapy. Stimulation of this pathway appears to add to an insulin stimulation of GLUT4 translocation and glucose transport, producing levels of stimulation that are greater than with single activator alone [76–79]. This suggests that there is some independence in the signalling inputs from insulin-stimulated kinases and principally the Akt signalling kinase. However, there is also cross-talk between the AMPK pathway and the Akt pathways and indications that there may also be some level of convergence of signalling at the common downstream signalling intermediates TBC1D1 and TBC1D4 [80,81].

To follow up on these observations on the levels of cell-surface GLUT4, we thought it useful to carry out kinetic studies on GLUT4 translocation in muscle tissue. We planned to use the biotinylated-hexoses to carry out these trafficking studies and avoid the technically difficult subcellular fractionation that had been used in adipocyte experiments. Such fractionation cannot be readily and reliably applied to the fibrous muscle tissue. We thought it feasible to resolve the biotin signature from biotin-tagged GLUTs either at the plasma membrane or at the intracellular membrane sites by quenching the plasma membrane signal with avidin (Figure 4b,c). We soon found that following labelling with bis-hexose photolabels such as Bio-LC-ATB-BGPA, the avidin did not react with the probes at all in intact cells. This biotinylated photolabel had been used only in experiments that measure the levels of cell-surface GLUT4. In this type of experiment, samples are solubilised in detergent and the GLUTs are unfolded and open when they bind streptavidin. In retrospect, the inability of these compounds to bind avidin added to intact cells is now quite understandable considering the deep cleft in the binding site that the crystal structures are now revealing.

**Figure 7.**
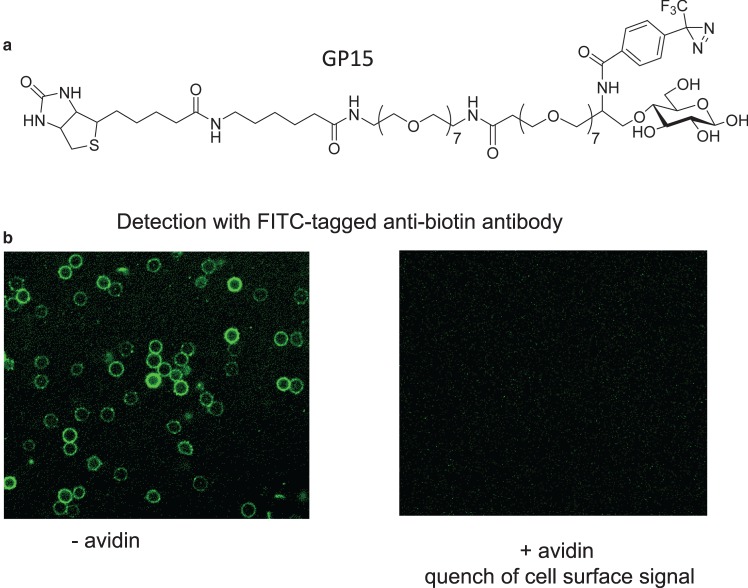
Extension of the linker in the biotinylated photolabel GP15 is necessary for interaction with avidin in intact cells. (**a**) GP15 is a glucose analogue substituted at C4-O with a photolabelling diazirine substituent and a very long polyoxyethylene spacer (of ∼70 Å). This spacer is necessary for interaction with avidin in intact cells (**b**). (**b**) Human erythrocytes have been labelled with GP15, a fluorescently labelled avidin was added to intact cells and fluorescence was detected by confocal microscopy. Data from ref. [82]. The necessity for a long spacer is presumably associated with the deep outside cleft of the GLUTs that is now evident in the crystal structures (Figure 3).

To address the problem of the deep binding cleft, Makoto Hashimoto and I therefore embarked on the preparation of a series of compounds with much longer chains and arrived at the compound that we call GP15. This has a sufficiently long chain to span the distance from the hexose-binding site in the GLUT and its external surface where avidin in solution can interact with the biotin tag. The GP15, with a linear projection of the polyethylene spacer, will span over 70 Å [82]. Note that each of the brackets (Figure 7a) is expanded seven times. We initially screened the series of long chain compounds on GLUT1 in erythrocytes. The surface biotin is detected with a fluorescent antibiotin antibody and the signal is quenched by avidin added directly to the intact cells. The labelling is also blocked by glucose competition at the labelling stage (Figure 7b).

With the availability of the GP15 compound, we devised an approach for measuring GLUT4 traffic, without using subcellular fractionation to separate the plasma membrane and intracellular pools of GLUT4. This is illustrated in Figure 4b,c. Basically, the experiment involves a pulse and a chase sequence. The idea is to firstly biotinylate cell-surface GLUT4 with GP15 and then allow it to move between compartments. Avidin added to the external solution then quenches the signal from the biotin that remains at the cell surface, while any internalised biotinylated GLUT4 escapes this quenching. The workup of the samples after this pulse and chase involves using immobilised streptavidin which captures any biotinylated GLUT4 that has internalised and has escaped the surface quenching. With this approach, we can design both exocytosis and endocytosis experiments. The former is easier to interpret kinetically as it involves the single rate constant for movement from inside to outside. The rate of internalisation is dependent on endocytosis and a component of recycling (Figure 4a) which contributes to the time course.

We have carried out this pulse and chase type of kinetic approach in intact adipocytes, in cardiomyocytes and skeletal muscle. The studies on the adipocytes confirmed data using the subcellular fractionation data and use of the tritiated photolabel, but in addition revealed aspects of the effects of catecholamines on insulin-stimulated GLUT4 translocation [83].

**Figure 8.**
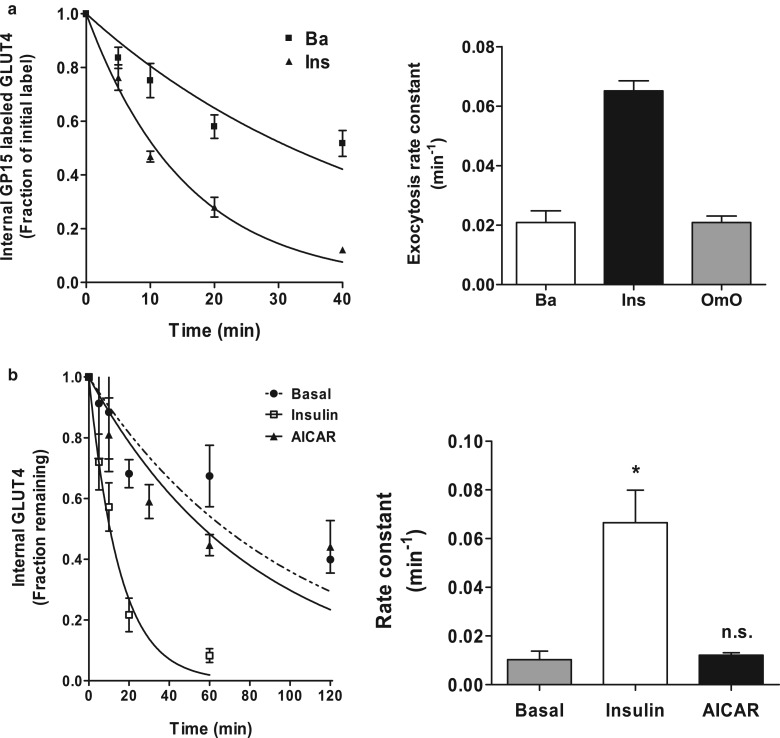
Insulin but not AMPK stimulation increases GLUT4 exocytosis in muscle. Exocytosis was determined using the biotin pulse, avidin chase protocol (Figure 4b,c) in which cell surface-biotinylated GLUT4 is internalised and then any cell-surface signal is quenched with avidin. This is followed in rat cardiac (**a**) and mouse epitrochlearis muscle (**b**). The exocytosis (loss of internal GLUT4, left panels) is quantified and rate constants are determined (right panels). Insulin stimulates the exocytosis rate constant above basal levels but AMPK activators, oligomycin in cardiomyocytes and AICAR in skeletal muscle do not. These data indicate that the insulin and AMPK stimulatory pathways (which both stimulate the levels of cell-surface GLUT4) do not converge at this step of GLUT4 translocation. Data are from refs [84,85].

Cardiac muscle cells are convenient to work with as a population of isolated cardiomyocytes can be obtained. We have compared exocytosis in basal and insulin-stimulated cardiomyocytes using the biotin pulse and avidin chase approach [79,84] (Figure 8a). In the basal state, most of the biotinylated GLUT4 that is internalised following the labelling pulse stays inside the cell and escapes the avidin quenching. However, following an insulin stimulation, the signal decreases more rapidly as the GLUT4 reaches the surface more rapidly. We have obtained a consistent value for the insulin-stimulated exocytosis rate constant (of ∼0.07 min^−1^), which is essentially the same as that which we obtained for adipocytes. Activation of AMPK through mitochondrial inhibition with oligomycin does not change the rate of exocytosis compared with the basal condition (Figure 8a), but instead causes a marked slowing of the whole time course for internalisation. This is consistent with a direct reduction in endocytosis associated with the AMPK-activating treatment [79,84].

It is more challenging to use the pulse-chase approach with skeletal muscle as a population of cells cannot be obtained and it is necessary to treat individual muscle strips to obtain each time point in a time-course assay. In collaboration with Juleen Zierath's group, we were able to carry out the GP15 labelling in rat epitrochlearis muscle [85] (Figure 8b). The internalised biotinylated GLUT4 is returned to the cell surface, and quenched by avidin, more rapidly when cells are treated with insulin than in basal cells. These experiments were difficult and there were large variations in observed signals. However, we fit the data to a very simple equation to obtain an exocytosis rate constant and the value we measured was consistent between experiments. The insulin-stimulated exocytosis rate constant in epitrochlearis muscle is 0.07 min^−1^ and is clearly significantly higher than that occurring in basal cells or AICAR-treated cells (Figure 8b). Both insulin and AICAR generate a similar level of steady-state cell-surface GLUT4 in skeletal muscle, but AMPK activation takes much longer to achieve this effect. The lack of a marked effect of an AMPK activation on exocytosis under these conditions suggests that the mechanism for the AICAR generation of high cell surface levels of GLUT4 differs from that in insulin-stimulated cells. This could involve an inhibition of endocytosis as observed in cardiomyocytes, as this would allow a slow build-up of cell-surface GLUT4 even when exocytosis occurs at the slow basal rate. Studies on phosphorylation of signalling intermediates, such as TBC1D1 and TBC1D4, suggest signalling convergence of insulin and AMPK signalling [80,86], but the kinetics of GLUT4 translocation suggest signalling from insulin and AMPK can have divergent effects on GLUT4 traffic. Changes in the AMP/ATP ratio that result from stimulation of the ‘exercise’ signalling pathway may have effects on endocytosis that are not present following an insulin treatment and are possibly independent of TBC1D1 and TBC1D4.

## Focusing on the membrane fusion step as the main regulated step in translocation

The similarity of the insulin-stimulated rate constant for exocytosis (0.07 min^−1^) in a range of tissues, with very different subcellular structures and architectures, suggests that the same fundamental process is occurring in adipocytes, cardiomyocytes and skeletal muscle. We concluded that fusion of vesicles at the plasma membrane may be rate-limiting for the overall GLUT4 translocation process and devised a chemical biology approach, involving the very long chain GP15, to examine this membrane fusion process.

**Figure 9.**
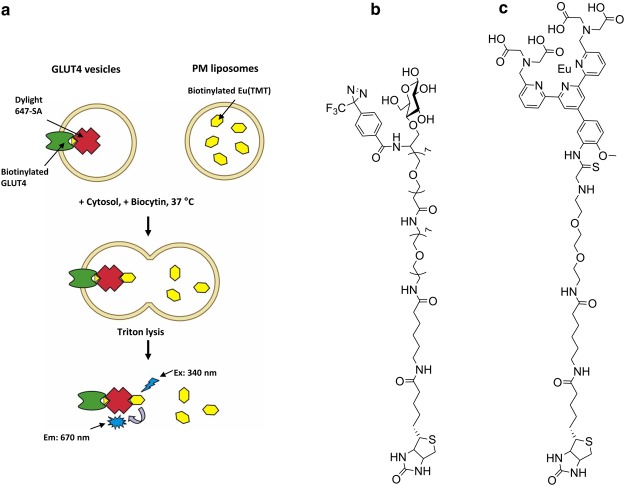
Components of a GLUT4 membrane fusion assay. The fusion assay is based on FRET between two, separately tagged, membrane compartments (**a**). The GLUT4 vesicle compartment is tagged with GP15 (**b**) onto which a fluorescent Dylight-streptavidin tetramer is conjugated. A fluorescent long chain-biotin europium Bio-Eu(TMT) (**c**) is enclosed within plasma membrane liposomes (formed from isolated plasma membrane and exogenous lipid). The components are incubated with cytoplasm that facilitates fusion that allows formation of a streptavidin complex that brings the two fluorescent molecules into close enough proximity for FRET (**a**). The complex is excited at 340 nm and the emission of the FRET signal is measured at 670 nm.

**Figure 10.**
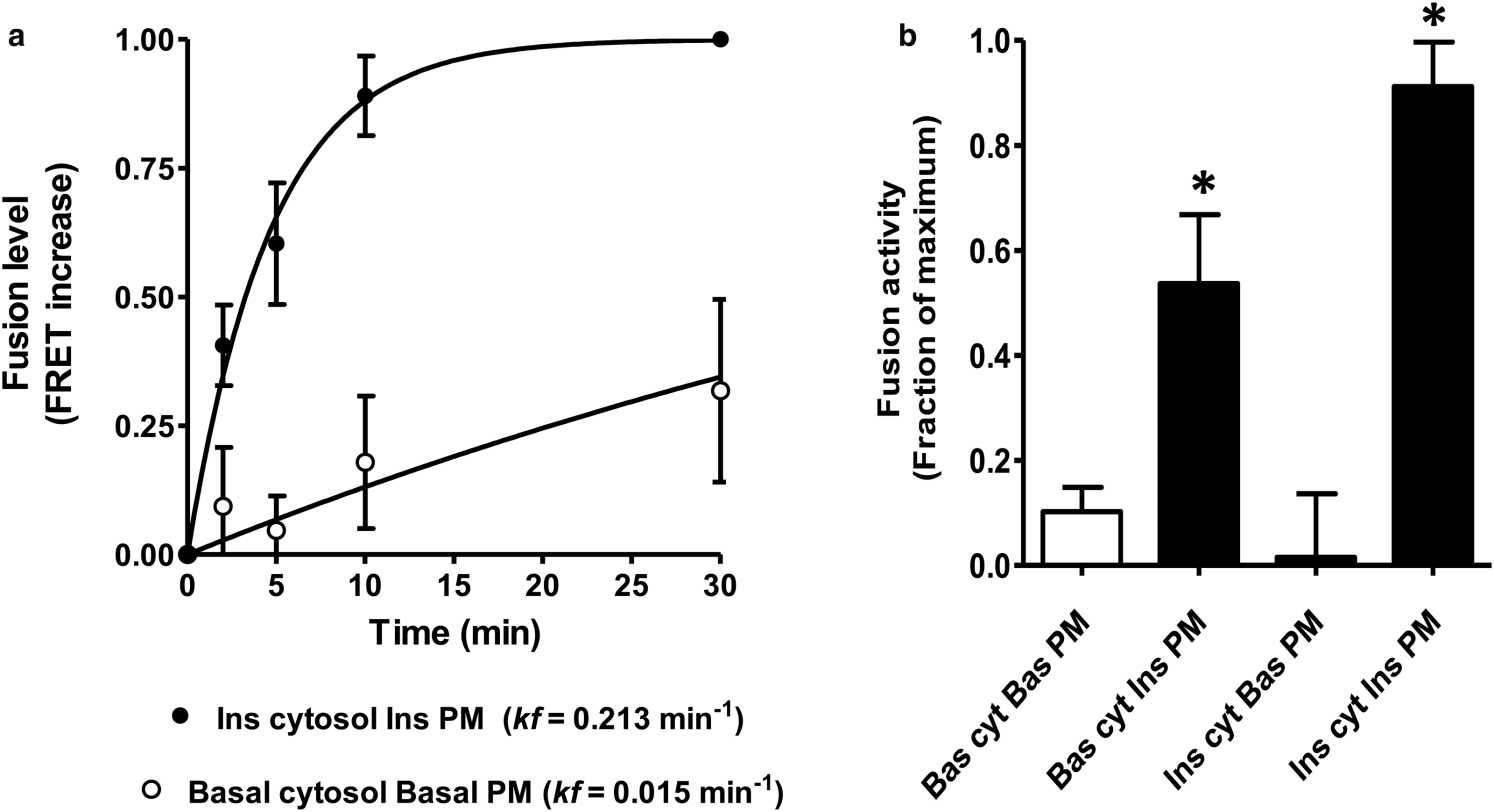
Insulin stimulation of GLUT4 vesicle fusion requires an insulin-activated plasma membrane. (**a**) A time-course assay reveals that insulin stimulates the fusion process in a completely cell-free system to an extent that fully capitulates the process of GLUT4 exocytosis in cells. The advantage of the cell-free approach is that components can be mixed in combinations that do not occur in cells. This allows a narrowing down of the critical components of the insulin-stimulated fusion. These component mixing experiments (**b**) reveal that insulin-activated plasma membrane can stimulate with the cytoplasm from the basal state fusion, whereas insulin-activated cytoplasm with basal plasma membrane is insufficient to stimulate fusion. In all cases, the GLUT4 vesicles are from the basal state. Data are from ref. [87].

To examine the fusion step in the absence of preceding steps in translocation, we decided that it would be necessary to work with cell-free components and then mix these components *in vitro* [87]. This allows the fusion step to be followed in the absence of processes such as release of vesicles from their internal reservoir compartment, or processes involving movement of vesicles along intact cell microfilaments and tubules. The pioneering work on cell-free fusion approaches described by Rothman and co-workers [88] guided our approach. A schematic illustrating our approach is illustrated in Figure 9. We require three main components for the assay (plasma membrane, GLUT4 vesicles and cell cytoplasm). Intact insulin-stimulated or basal adipose cells were tagged by the GP15 biotinylated photolabel and further conjugated to Dylight 647 streptavidin. Subsequently, GLUT4 vesicles were obtained from these biotinylated and fluorescently tagged samples. In separate preparations, we isolated plasma membrane from insulin-stimulated or basal cell adipose cells and then reconstituted these into liposomes. While forming these liposomes, we trapped a biotin ligand that has a europium chelate attached (Bio-Eu-TMT, Figure 9c). To measure fusion activity, we mix these two sets of vesicles in the presence of separately prepared cytoplasm and incubate at 37°C. We stop the reaction with a triton-lysis step at intervals. When the two sets of vesicles fuse, the Bio-Eu-TMT (initially from the plasma membrane liposomes) now has access to the sites in the streptavidin tetramer (initially from the GLUT4 vesicles). The Bio-Eu-TMT is now close enough to the Dylight 647 (both ligands are on the streptavidin complex) for FRET to occur. We excite the Bio-Eu-TMT at 340 nm and this emits at a wavelength that excites the Dylight. This then emits at 670 nm. We do not have to remove excess Bio-Eu-TMT as this will not undergo FRET as it not close enough to the unconjugated Dylight streptavidin. We have measured the increase in FRET activity with time as the contents of two sets of vesicles fuse together (Figure 10a). The time course reveals that the assay recapitulates both the magnitude and time course of the insulin response in intact cells. The rate constant is increased by ∼10-fold. Importantly, the rate constants that we calculate are similar to those for stimulation of GLUT4 exocytosis which suggests that the fusion step determines and limits the kinetics of the whole exocytosis process. The data are highly consistent with the TIRF experiments carried out by the groups of Sam Cushman, David James and Tim McGraw [89–91] who published roughly at the same time and showed, using microscopy, that fusion activity was increased by insulin.

An advantage of the *in vitro* fusion assay is that the separate components of the assay can be manipulated in a manner that cannot readily be studied by other methods. For example, if we mix cytoplasm and vesicles from basal cells with the insulin-stimulated plasma membrane, then this is sufficient to drive the fusion reaction (Figure 10b). An insulin-stimulated cytoplasm is insufficient to drive fusion of vesicles if the plasma membrane and GLUT4 vesicles are derived from basal cells. These data suggested that we had identified that (in the absence of intact intracellular compartments), fusion is the key regulated step in GLUT4 translocation and that the plasma membrane has to be in an insulin-activated state for fusion to occur [87]. We think that fusion alone is sufficient to account for most of the insulin-stimulated translocation that is observed in insulin-responsive cells. However, there are several groups with data suggesting, that in addition to stimulating the final plasma membrane fusion process involved in exocytosis, insulin acts at intracellular sites [69,71,92–95]. It is now clear from studies on the cell biology of GLUT4 that this vesicle cargo travels through multiple compartments and that perturbation of any of these can influence translocation. However, the extent to which movement through these compartments is influenced by insulin activation steps is still incompletely resolved and further modelling of these processes, incorporating the likelihood that some compartments can be saturated and reach a maximum filling with GLUT4, is still required.

Rab proteins and the upstream Rab-GAP proteins TBC1D1 and TBC1D4 have been strongly implicated in GLUT4 traffic [96–98]. The site of action of these proteins and the ways in which they influence GLUT4 traffic are being widely investigated. These are of interest as their activity can link insulin signalling involving protein phosphorylation with membrane traffic which is directed by the Rab proteins. For example, the phosphorylation of the Rab-GAPs can influence either the loading of Rabs with GTP or the extent to which the GTP/GDP Rabs can bind to GLUT4 vesicles [99,100].

Rather than discussing these ongoing studies on GLUT4 membrane traffic and its control by Rab GTPases at length, I thought I would continue here with the chemical biology theme and describe a new label which is a GTP photoaffinity analogue. The aim of the course was to label the Rab proteins and potentially identify intermediates between insulin signalling and GLUT4 traffic.

## Progress towards identifying key Rab proteins involved in GLUT4 subcellular traffic

We have made several Rab photolabels including molecules substituted on the terminal phosphate or on a terminal thiol in GTP-γ-S. The structure that seems to work best is the one with this bulky photolabel moiety substituted on the ribose. Examination of crystal structures of a GTP analogue bound to the Rabs [101] reveals that the guanine and phosphate moieties are quite buried, but the ribose hydroxyls at C2-OH and C3-OH project into the solution.

**Figure 11.**
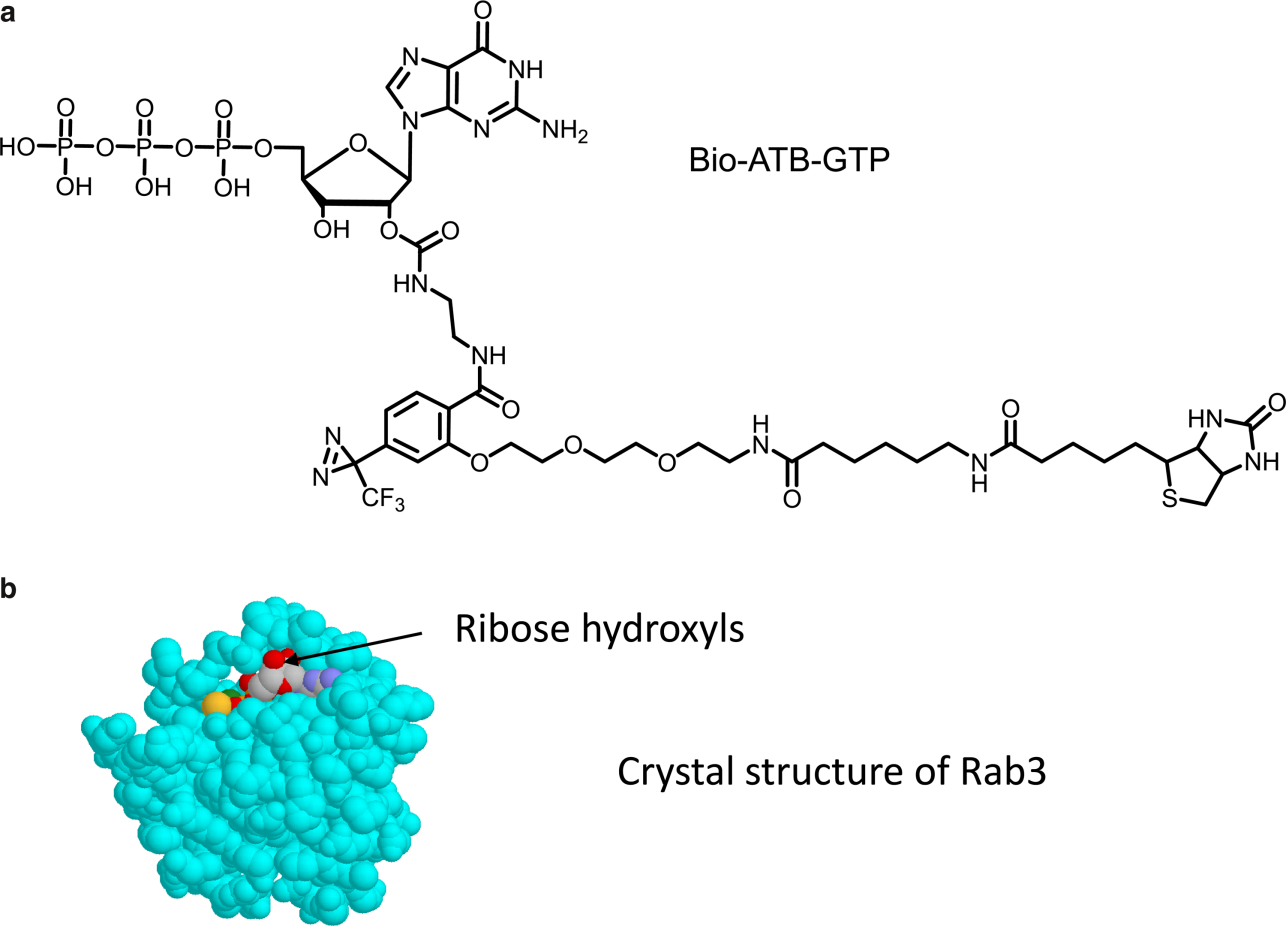
A new reagent for investigation of small G-proteins. A new G-protein labelling reagent Bio-ATB-GTP (**a**) has been developed for studying the extent of insulin activation of these proteins. The substitution onto GTP was through the ribose hydroxyls as the crystal structures of G-proteins (**b**) revealed that these were more exposed on the surface of the protein that the guanine base or the ribose phosphates.

**Figure 12.**
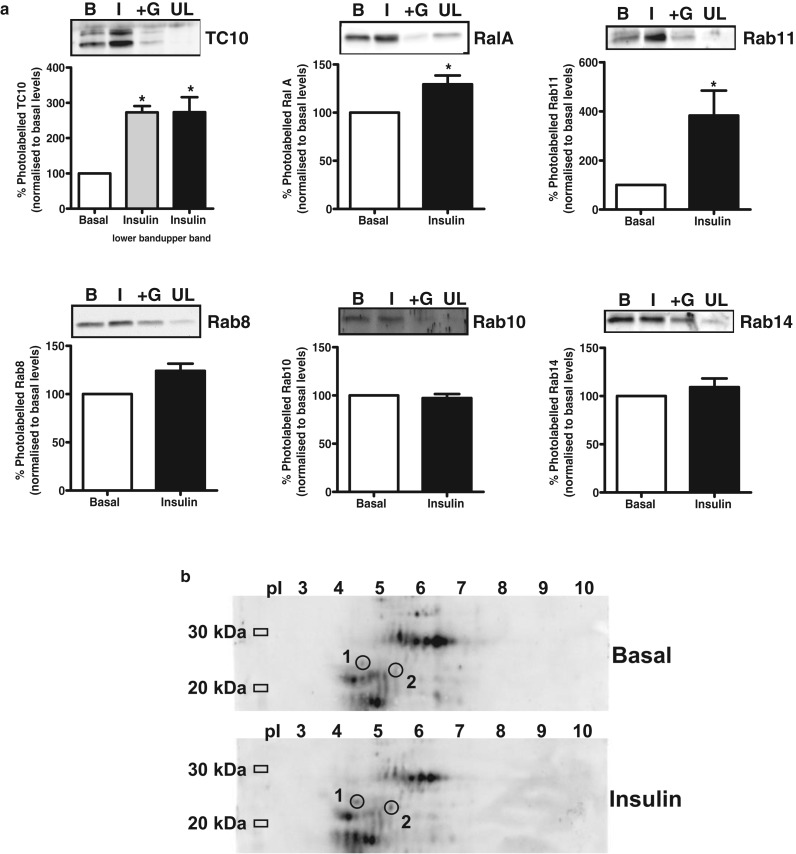
Approaches used for detection of insulin-activated Rab proteins. (**a**) A targeted approach for the determination of the extent of insulin activation of G-proteins of interest is used. Membranes from rat adipocytes are labelled with Bio-ATB-GTP, solubilised and precipitated on immobilised streptavidin, resolved on SDS–PAGE gels and then blotted with target-specific antibodies. Background signal is determined by competition with GTP (+G) and an unlabelled membrane sample (UL). TC10, RalA and Rab11 (top row of panels) have significantly higher labelling in membranes from insulin-treated cells (I) than basal cells (B), but Rab8, Rab10 and Rab14 (bottom row of panels) do not. In (**b**), the labelled membranes are resolved on 2D gels and G-proteins detected by streptavidin peroxidase and identified by mass spectroscopy. Both Rab 3b (1) and Rab11a (2) have higher levels of labelling in membranes from insulin-stimulated compared with basal cells. Data (apart from the Rabs 8, 10 and 14 blots) are from ref. [102].

The molecule we use (Bio-ATB-GTP, Figure 11a) has a side-chain attachment to the ribose which incorporates biotin plus photoreactive diazirine moieties. This combination of substituents is similar to those used with the hexose photolabels. The approach is to cross-link to the protein, in this case to GTPases, and then use the biotin tags for subsequent target isolation [102]. There are several subsequent approaches that we could take for identifying Rab proteins. First, we can use a targeted approach in which we label membranes and then precipitate the tagged proteins with immobilised streptavidin and finally blot with a target-specific antibody. Figure 12a illustrates some examples of Bio-ATB-GTP labelling of some potential targets of insulin action. Blotting of Bio-ATB-GTP-labelled membranes in the insulin and basal conditions for TC10, Ral1A and Rab11 reveals that each of these G-proteins has increased labelling that is dependent on insulin signalling. Excess GTP competition experiments and comparisons with unlabelled membranes (to detect any non-specific carry through during the precipitations) are used as control lanes on the gels (Figure 12a, top panels). The labelling of some Rab proteins, such as Rab8, Rab10 and Rab14 that have been implicated in GLUT4 traffic and are substrates for TBC1D1 and TBC1D4, is not increased in the membranes from insulin-treated, compared with basal cells (Figure 12a, bottom panels). Perhaps, the technique is not sensitive to insulin activation of these Rab proteins and other techniques may be needed to demonstrate that insulin action directly influences the labelling of these TBC1D1/TBC1D4 targets.

A more generic approach for identification of insulin-regulated Rabs is to label cell membranes from basal and insulin-stimulated cells with Bio-ATB-GTP and then separate proteins on 2D gels and identify spots that are differentially labelled in the insulin-stimulated compared with the basal condition (Figure12b). The labelled proteins are detected with streptavidin peroxidase and any spots of interest can be analysed by mass spectroscopy. Some detected spots increase, and some decrease, in intensity in the insulin-treated sample. Rab3b, which was identified from Spot 1 (Figure 12b), has increased labelling in the insulin sample. Rab11a was identified from Spot 2. The increased labelling of Rab11 that is identified using the generic 2D gel approach is consistent with the data from the targeted approach (Figure 12a).

The generic labelling approach has great potential utility, but there are several limitations. Although the spots identified using the label appear quite discrete, total protein staining of all the proteins in the sample (labelled and non-labelled) is extensive and widespread. There is protein crowding in many areas of the gel that are of interest, and this limits the ability to discretely cut out the protein of interest from the gel. Fortunately, Rab3b and Rab11a (spots 1 and 2, Figure 12b) are in relatively clear areas of the gel and isolating the discrete protein is possible. Another problem is that the blotting using streptavidin-peroxidase is very sensitive and, in some cases, the intensive staining is not matched by useful levels of isolatable protein. In future, improvements in the technique and the sensitivity of mass spectroscopy instruments may lead to the identification of a wider range of insulin-activated GTPases.

Once identification of an insulin-regulated target had been made, Francoise Koumanov, in my laboratory, followed up by expressing a flag-tagged version of Rab3b in adipocytes. The use of dominant negative and constitutively active Rab3b constructs implicated Rab3 in aspects of regulation of GLUT4 vesicle interaction with the plasma membrane. In addition, we have examined Rab3-interacting molecules that may be involved in the Rab3 interaction with the GLUT4 translocation process. These include the effector Noc2 that is present in the plasma membrane in the basal state and dissociate from the plasma membrane following an insulin stimulation [102].

Rather than describing these new biochemical and cell biological data specifically on Rab3, I thought it is more appropriate in this lecture/review to look to the future in terms of the applications of chemical biology to GLUT research. Further research is being carried out in many laboratories on the many G-proteins that may link to insulin signalling kinases and have roles in GLUT4 traffic. Some of these studies are described in leading papers and reviews [103–107].

## Looking to the future of chemical biology approaches to studying GLUT structure and function

With the availability of the new crystallographic data on the GLUT family of proteins, it is likely that new reagents and substrate analogues can be developed that are specific for individual GLUTs. It has long been clear that pharmaceutical control of glucose transport is desirable, particularly in the cancer biology field [108–110]. However, as glucose uptake is so essential to all cells of the body, a generalised knockdown of GLUT activity is unlikely to be useful. In addition, preventing glucose entering cells may, in some cases, be desirable but could potentially lead to hyperglycaemia which is clearly undesirable. By using the more detailed structural information that is now available for the GLUT proteins, the design of new reagents that are specific for individual GLUT isoforms and are useful in therapies may be feasible? There are now increasing numbers of chemical biology studies in the literature that use chemical library screening to identify GLUT inhibitors [109]. These are often based on cell screens used in conjunction with *in silico* screens that involve the docking of potential inhibitors on computer models of known crystal structures of the GLUTs.

A GLUT1-specific inhibitor might be useful as this GLUT isoform is known to be up-regulated in many cancers and is associated with the dependence of cancers on glycolytic metabolism, the Warburg effect [111,112]. One of the many typical screens has been described by Siebeneicher et al. [110]. This group initially screened 3 million compounds and then took the lead compound and further chemically modified it to end up with the compound Bay 876 that has low nM affinity for GLUT1 but much less affinity for the other GLUTs tested. The Siebeneicher group refined their GLUT inhibitors by obtaining a crystal structure of GLUT1 with bound cytochalasin B (the specific inhibitor of GLUTs that kinetic and photoaffinity labelling studies had revealed to bind exclusively at the inward-facing site [7,35]). A range of highly modified peptide-like compounds (Glut-i1 and Glut-i 2) that bound to the same inward-facing site as cytochalasin B were studied in detailed crystal structures with bound ligands [57]. Of course, there is no information yet on the range of off-target interactions that these screening-derived compounds might have. However, there seems to be real potential for GLUT-specific targeting and possibly GLUT inhibitor-based therapies.

There is also great potential (and possibly less toxicity) in the use and design of inhibitors based on naturally occurring compounds such as the polyphenols. Compounds such as the polyphenolic compounds derived from green tea are known to have therapeutic value. This is thought to be due to their ability to quench free radicals, but these compounds are also very powerful inhibitors of GLUT1-mediated glucose transport [113]. Phloretin and phloridzin are derived from apple leaves, bark and seeds. Phloretin is a well-known GLUT inhibitor that many have used in glucose transport time-course assays, where a powerful inhibitor is required to terminate the transport. Phloridzin has higher specificity for the sodium-dependent glucose transporters (the SLGTs) than the GLUTs. It has been used experimentally as a renal glucose reabsorption inhibitor that lowers blood glucose and leads to glucosurea [114]. A derivative of phloridzin has been developed as a blood glucose-lowering compound by Bristol Meyer Squib and AstraZeneca. This now has FDA approval for use with metformin in the treatment of type 2 diabetes [115]. So, can a combination of a GLUT isoform-specific inhibitor (that would tend to raise blood glucose) plus a phloridzin-like compound (to increase renal loss of glucose) be useful in cancer treatment?

**Figure 13.**
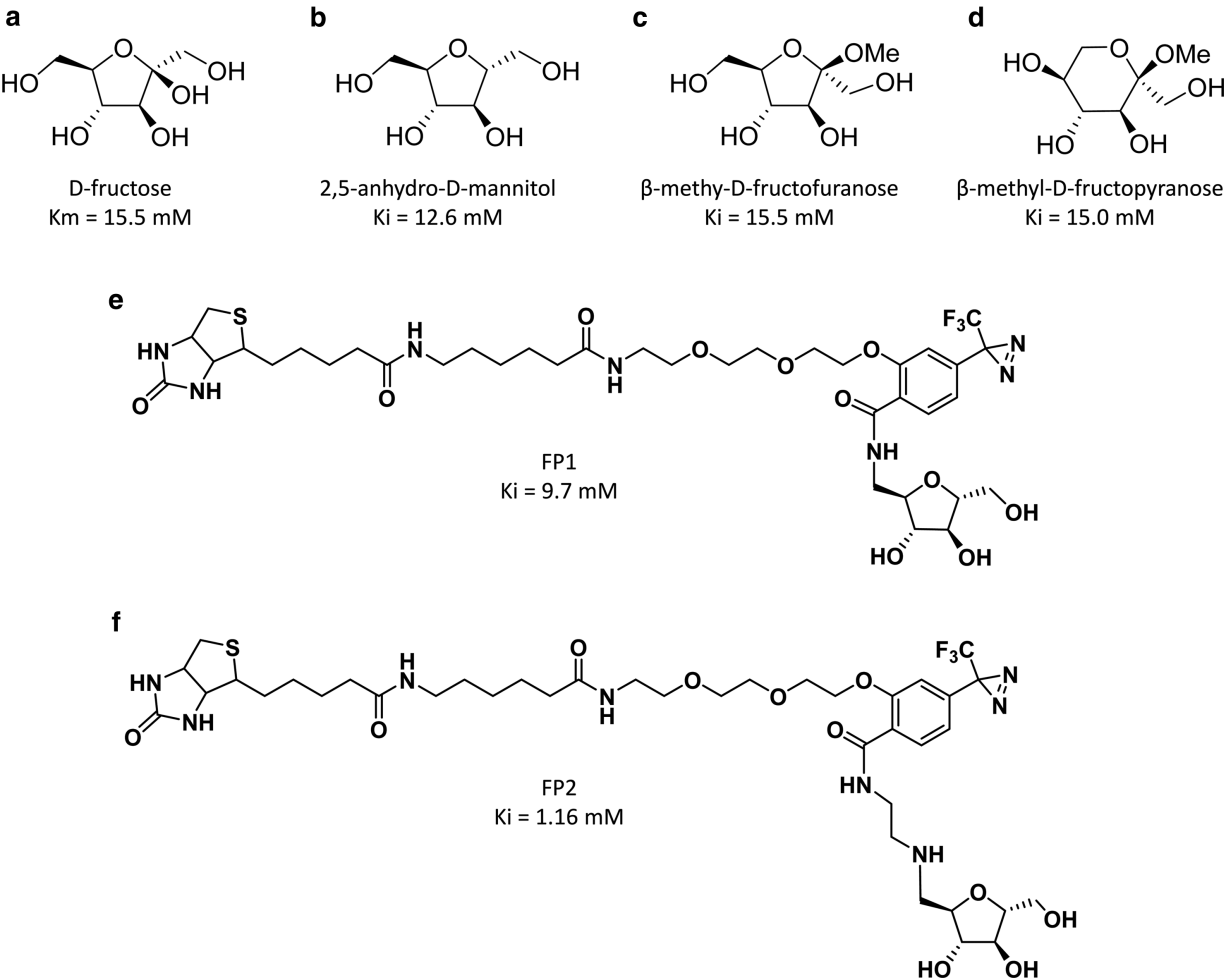
Analogues used to study the fructose transporter GLUT5. Fructose (**a**) can adopt α and β of fructofuranose and fructopyranose in solution (in a ratio αP:βP:αF:βF <1%:75%:4%:21%). GLUT5 interacts with both forms as the closed-ring structures (**b**–**d**) are good inhibitors of transport with *K*_i_ values comparable with the *K*_m_ values of d-fructose. The C2-β-*O*-methyl fructofuranoside and fructopyranoside both have ∼5-fold higher affinity than the corresponding C2-α-*O*-methyl compounds. The closed-ring 2,5-anhydro-d-mannitol (2-deoxy-d-fructose) (**b**) is a useful compound for synthesis of d-fructose biotinylated fructose photolabels FP1, FP2 (**e,f**). As in the GLUT1–4 photolabelling compounds, the spacer distance between the fructose and benzoyl-diazirine group is critical for affinity enhancement; compare fructose (**a**) with FP1 (**e**) and FP2 (**f**). Data are from refs [20,120].

GLUT5 is known to be up-regulated in cancer of some tissues [109,116,117] and fructose (derived from all the sucrose we consume) probably does more harm than good in the body as it can contribute toward non-alcoholic fatty liver disease [118]. In addition, fructose is more rapidly converted to advanced glycation end products than glucose, but the significance of this fructation is debated [119]. So, can GLUT5 inhibition be specifically targeted by analogues? Using a range of fructofuranose and fructopyranose derivatives, we have mapped features of the binding site in GLUT5. Fructose adopts α and β forms of fructopyranose and fructofuranose in solution (αP:βP:αF:βF <1%:75%:4%:21%). GLUT5 probably mainly interacts with β-forms since both ring-closed furanose and pyranose derivatives are good inhibitors of transport with *K*_i_ values comparable with the *K*_m_ of d-fructose (Figure 13a–d). C2-β-*O*-methyl-d-fructofuranoside (Figure 13c) and C2-β-*O*-methyl-d-fructopyranoside (Figure 13d) both have ∼5-fold higher affinity than the corresponding C2-α-*O*-methyl compounds [20]. 2,5-anhydro-d-mannitol (2-deoxy-d-fructofuranose, Figure 13b) is a particularly useful GLUT5 analogue that retains a furanose ring shape, as it cannot ring open and therefore cannot form anomers. We have found that photoaffinity labelling compounds (FP1 and FP2, Figure 13e,f), which are based on 2,5-anhydro-d-mannitol, combine well with GLUT5 when this is expressed in CHO cells [120], but so far, these analogues have not been extensively tested on GLUT5-expressing tissues. Fluorescent GLUT5 probes based on the 2,5-anhydro-mannitol ring have also recently been designed [121]. The potential for targeting the unique specificity of the fructose transporters is therefore good [108,116]; particularly now there are good crystal structures of GLUT5 [61]. Detailed modelling may be required to design fructose analogues that are transported only by GLUT5 and not by GLUT2 (which has significant affinity for fructose) [122,123].

Finally, can GLUT4 be usefully targeted by inhibitors of transport? This would seem on the face of it to be a very bad idea as inhibition of GLUT4-mediated glucose transport into the major tissues of fat, muscle and heart would lead to transient hyperglycaemia, and this would have to be counteracted by increasing renal clearance of the excess circulating glucose in the longer term. But would seeking health benefits from increasing GLUT4-mediated glucose uptake and insulin sensitivity be such a panacea either?

The consequences of GLUT4 knockout in humans are complex [124]. However, homozygous knockout of GLUT4 in mice is not lethal [125], while heterozygous GLUT4 knockout mice do develop diabetic histo-pathologies but are lean. We have partially explored this issue in a simpler model system, the nematode worm (*Caenorhabditis elegans*). In collaboration with Adrian Wolstenholme, we cloned the *C. elegans* GLUT, which we called FGT1 (Facilitative Glucose Transporter 1) [126]. The presence of high glucose in the medium is known to reduce lifespan in these worms [127,128]. It halved the lifespan in our experiments. Conversely, it is well established that knocking out the receptor for insulin-like peptides (Daf2) and the downstream PI3-kinase (Age-1) in *C. elegans* can prolong lifespan [129,130]. We therefore examined the effects of knockdown of glucose uptake via FGT1. Knockdown of FGT1 in a normal glucose medium of wild-type worms increased lifespan to an extent which was comparable (but not additive) with that occurring in Daf2 or Age1 knockout mutant worms. In addition, polyphenolic compounds, which have multiple effects but are known glucose transport inhibitors, can prolong lifespan in *C. elegans* [131]. Similar lifespan extending effects of knockdown of insulin signalling are also seen in fruit flies and in mice [132]. Some of these effects may be related to reductions in stored calories. These comparisons with simple organism lifespan over-simplify the role of GLUT4 in human physiology and one would not really want to develop a GLUT4-specific inhibitor solely based on these considerations. However, if such an inhibitor were available, it would be interesting to study any potential health benefits.

So, in summary, it seems that designing selective drugs for individual GLUTs is likely to be feasible in future, but the use of any such drug in a therapeutic role would have to be done very carefully. There is a lot we still need to know about both the chemistry and biology of GLUTs and I look forward to seeing how these lines of research will develop in future.

## Abbreviations

BMPA: bis-mannose photolabel, ATB
FGT1: Facilitative Glucose Transporter 1
GLUTs: glucose transporters
